# Molecular docking and statistical optimization of taurocholate-stabilized galactose anchored bilosomes for the enhancement of sofosbuvir absorption and hepatic relative targeting efficiency

**DOI:** 10.1080/10717544.2020.1787557

**Published:** 2020-07-02

**Authors:** Marianne Joseph Naguib, Amira Moustafa Kamel, Ahmed Thabet Negmeldin, Ahmed Hassen Elshafeey, Ibrahim Elsayed

**Affiliations:** a Department of Pharmaceutics and Industrial Pharmacy, Faculty of Pharmacy, Cairo University, Cairo, Egypt; b Polymer and Pigments Department, National Research Center, Cairo, Egypt; c Department of Pharmaceutical Organic Chemistry, Faculty of Pharmacy, Cairo University, Cairo, Egypt; d Department of Pharmaceutical Sciences, College of Pharmacy and Thumbay Research Institute for Precision Medicine, Gulf Medical University, Ajman, UAE

**Keywords:** Sofosbuvir, liver targeting, bilosomes, bile salts, taurocholate, galactose, molecular docking

## Abstract

The work aimed to improve both absorption and hepatic availability of sofosbuvir. Bilosomes and galactose-anchored bilosomes were investigated as potential nanocarriers for this purpose. Sofosbuvir is a class III drug with high solubility and low permeability. Thus, the drug entrapment into lipid-based galactose-anchored carriers would enhance drug permeability and improve its liver availability. The galactosylated taurocholate was designed and synthesized based on molecular docking studies, where both galactose and taurocholate molecules were connected in a way to avoid affecting crucial interactions and avoid steric clashes with their cellular uptake receptors. The suggested nano-carriers were prepared using a thin-film hydration technique with sodium taurocholate and span 60 as stabilizers. The prepared formulae were statistically optimized using a central composite design. The optimized plain and galactosylated formulae, composed of SAA to drug ratio of 1:1 w/w and sodium taurocholate to span ratio of 10:1 w/w, have a vesicular size, zeta potential and entrapment efficiency in the range of 140–150 nm, −50 mV and 85%, respectively. The optimized formulae were lyophilized to increase their physical stability and facilitate accurate drug dosing. In vivo results showed that Sofosbuvir availability in the liver was significantly increased after oral administration of the plain and the galactosylated bilosomal formulae when compared to the oral drug solution with relative targeting efficiencies (RTIs) of 1.51 and 3.66, respectively. These findings confirmed the hypothesis of considering the galactosylated bilosomes a promising nanocarrier to efficiently target sofosbuvir to the liver.

## 1. Introduction

Hepatitis C is considered as one of the critical silent killer diseases with fatal long-term complications including liver cirrhosis and cancer. The disease remains hidden in most of the patients with increased spreading worldwide, 160–170 million patients, and 350,000 annual deaths with liver disease arising as a result of this infection(Lawitz et al., 2013; European Association for Study of Liver, 2014).

Sofosbuvir is an analog to uridine nucleotides. It was first synthesized in 2007 and approved by the FDA in 2013 to be administered in a dose of 400 mg per day in combination with ribavirin in the form of oral tablets for the treatment of genotypes 2 and 3 (Donaldson et al., 2015). Moreover, interferon was approved to be added to the sofosbuvir/ribavirin mixture to form a triple therapy for controlling genotypes 1 and 4 (Lawitz et al., 2013). Sofosbuvir is combined also with other novel antivirals, including simeprevir, daclatasvir and velpatasvir, to give a cure percentage ranged between 30% and 97% based on the involved cases (Buti et al., 2017; Saint-Laurent Thibault et al., 2017; Younossi et al., 2017). According to the biopharmaceutics classification system, sofosbuvir is considered as class III drug with high solubility and low permeability (Kirby et al., 2015). Its solubility is equal, or more than 2 mg/mL and its partition coefficient is about 1.6. Moreover, it is a weak acid with pKa of 9.6 and so, it remains unionized throughout the gastrointestinal tract, regardless of the surrounding physiological pH (Amidon et al., 1995). Chemically, its molecular formula is C_22_H_29_FN_3_O_9_P and molar mass equals 529.45 g/Mol (Bhatia et al., 2014). Sofosbuvir is an inactive prodrug, activated in the liver into the active form 5′ triphosphate nucleotide (GS-461203) which inactivated again into GS-331007 by dephosphorylation (Kirby et al., 2015). After oral administration, 36.4% of the administered dose is absorbed through the portal circulation with 74% hepatic extraction leading to 26.94% hepatic availability of the administered dose (Rodríguez-Torres, 2013; Kirby et al., 2015; Cuenca-Lopez et al., 2017). Sofosbuvir is a substrate to P-glycoprotein (P-gp) which leads to its efflux from the GIT membrane cells and hepatocytes as well as counteracting its cellular internalization (Shen et al., 2019). In the case of hepatitis C infection, P-gp expression is upregulated 2–5 times in terms of mRNA and 20 times in terms of protein which could significantly enhance sofosbuvir efflux and reduce its cellular uptake (Thakkar et al., 2017). Moreover, Sofosbuvir is extensively distributed throughout the body with a 127-liter volume of distribution leading to multiple side effects, namely, fatigue, headache, nausea, insomnia, itching, anemia, weakness and rash and keeping its level in a minimal range in the target organ (Bhatia et al., 2014; Reznik & Ashby, 2017). Thus, it can be deduced from the previously mentioned facts that sofosbuvir is considered as a good candidate for liver targeting that would enhance its availability, residence time in the liver and efficacy. Nanovesicles have been used for many years as drug carriers to enhance oral absorption (Banerjee, 2001). Liposomes are the most common and most studied nanovesicles. Later, several modifications have been applied to improve the physical characteristics and enhance the drug delivery efficacy of liposomes, including niosomes, ethosomes, transfersomes, bilosomes, glycerosomes and lipotomes (Elkasabgy et al., 2014; Ahmad et al., 2017; Zhao et al., 2017; Attia et al., 2018; Manconi et al., 2018; Naguib et al., 2020). Bilosomes are modified liposomes with the incorporation of bile acids or bile salts (Parashar et al., 2019). They were found to be effective carriers for enhancing corneal and transdermal permeation, in addition to oral absorption (Al-mahallawi et al., 2015; Abdelbary et al., 2016). Moreover, bilosomes were used to stabilize drugs and vaccines after oral administration by protecting them against digestive enzymes (Wilkhu et al., 2013; Aburahma, 2016). On the other hand, bile salts were previously investigated for their ability to specifically deliver the drug to the liver (Chen et al., 2017; Pathak et al., 2018). They are liver-produced amphipathic molecules that are required to facilitate the absorption of cholesterol, fat-soluble vitamins and lipids in the intestine (Sharma et al., 2016). Farnesoid X receptor (FXR), G protein-coupled bile acid receptor 1 (TGR5) and the apical sodium-dependent bile acid transporter (ASBT) were recently identified as bile salts receptors in both liver and intestine (Pathak et al., 2018). The presence of these receptors could provide a way for the specific pick-up of bilosomes to achieve liver targeting. Also, galactose was previously utilized to chemically modify different types of nanovesicles to enhance liver targeting of the encapsulated drug (Maepa et al., 2018; Pathak et al., 2018; Diaz-Galvez et al., 2019; Patil et al., 2019). It has specific receptors on the hepatocytes (Asialoglycoproteins: ASGPR) and consequently, it acts as a vector for the active targeting of the drug encapsulated in the galactosylated nanovesicular carrier (Tanaka et al., 2017; Maepa et al., 2018). In addition to the capability of the span containing nanocarriers to inhibit efflux pumps (P-gp) that acts as a barrier against drug accumulation in hepatocytes (Kaur et al., 2016; Nour et al., 2016).

To the best of our knowledge, no trials have been reported for the synthesis of galactosylated taurocholate neither the use of galactose-anchored bilosomes as potential carriers for liver targeting. Thus, the present work aimed to formulate bilosomes, plain and galactose-anchored, and investigate their capabilities to target sofosbuvir to the liver. Central composite design and desirability function were employed to optimize sofosbuvir bilosomes with minimized vesicular size and polydispersity index, maximized zeta potential and encapsulation efficiency. The optimized formulation was then prepared using galactosylated taurocholate. Both formulae were lyophilized to improve their physical stability then assessed using morphological characteristics, *in vitro* release and *in vivo* liver uptake. The liver targeting ability of the developed bilosomes were judged in terms of Cmax, Tmax, AUC, percent hepatic availability, drug targeting index and relative targeting ability Increasing the hepatic availability of sofosbuvir can increase drug efficacy and decrease its side effects. To our knowledge, galactose-anchored bilosomes were not previously investigated as potential carriers for liver targeting.

## 2. Materials and methods

### 2.1. Materials

Sofosbuvir was a gift sample from Pharco Pharmaceutical Co., Alexandria, Egypt. Sodium taurocholate (STC), l-α-Phosphatidylcholine (PC), span 60 (S60) and galactose were purchased from Sigma-Aldrich, St. Louis, MO, USA. The remaining chemicals and solvents were of analytical grade and utilized without further purification.

### 2.2. Preparation of sofosbuvir bilosomes

Bilosomes were prepared using the thin film hydration technique (Bangham et al., 1965). Briefly, sofosbuvir (100 mg), STC, PC and S60 were accurately weighed, dissolved in 10 mL mixture of methanol: methylene chloride, in a ratio of 1:3 v/v, and transferred into 250 mL round-bottom flask. Under vacuum, the mixture of the organic solvents was evaporated using the rotary evaporator (Rotavapor, Heidolph VV 2000, Burladingen, Germany) rotating at 80 rpm for 30 min at temperature 50 °C. The wall-assembled thin film was hydrated using 10 mL double-distilled water under normal pressure. Finally, the prepared bilosomes were sonicated in an ultrasonic bath (Model SH 150-41, PCI Analytics Pvt. Ltd, Mumbai, India) for 3 min to avoid aggregation (Mishra et al., 2007).

### 2.3. Statistical design

A central composite design was utilized to study the effect of the formulation variables on the characteristics of the prepared bilosomes using Design-Expert^®^ 7 software (Version 7, Stat-Ease Inc., Minneapolis, MN, USA). Two independent factors were studied as follows: surfactants (SAA) to drug ratio (X_1_) and STC to S60 ratio (X_2_), as shown in Table 1. The traced responses were the vesicular size (VS, Y_1_), polydispersity index (PDI, Y_2_), zeta potential (ZP, Y_3_) and encapsulation efficiency (EE, Y_4_). The composition of the nine prepared formulae is displayed in Table 1.

**Table 1.** Experimental runs, independent variables, and measured responses of the central composite experimental design for sofosbuvir bilosomal formulae.

Formula code	X_1_: SAA/Drug ratio	X_2_: STC/S60 ratio	Y_1_: VS (nm)	Y_2_: PDI	Y_3_: ZP (mV)	Y_4_: EE (%)	DL (%w/w)
B1	1	1	177.5 ± 3.7	0.45 ± 0.03	−30.4 ± 2.5	79.3 ± 5.1	15.9 ± 1.1
B2	1	5.5	191.0 ± 4.0	0.77 ± 0.03	−39.2 ± 1.8	58.7 ± 3.4	11.7 ± 1.5
B3	1	10	149.0 ± 2.8	0.27 ± 0.01	−49.5 ± 3.3	85.8 ± 3.7	17.2 ± 1.9
B4	3	1	200.2 ± 3.3	0.37 ± 0.02	−30.6 ± 1.2	62.1 ± 4.8	8.9 ± 0.8
B5	3	5.5	192.0 ± 5.1	0.66 ± 0.04	−42.2 ± 1.9	81.8 ± 3.3	14.2 ± 1.4
			189.3 ± 4.4	0.72 ± 0.04	−40.1 ± 3.7	57.4 ± 2.0	8.2 ± 0.5
			201.3 ± 1.6	0.64 ± 0.02	−42.5 ± 0.7	63.5 ± 1.9	9.1 ± 1.0
			225.2 ± 6.9	0.70 ± 0.01	−41.5 ± 2.8	12.6 ± 0.5	1.4 ± 0.2
			202.8 ± 3.6	0.67 ± 0.01	−41.3 ± 3.9	59.6 ± 4.1	8.5 ± 0.3
B6	3	10	182.3 ± 1.4	0.35 ± 0.02	−48.2 ± 3.5	13.9 ± 1.1	2.0 ± 0.3
B7	5	1	238.1 ± 1.1	0.61 ± 0.05	−37.1 ± 1.0	9.5 ± 0.3	1.1 ± 0.2
B8	5	5.5	226.3 ± 5.8	1.00 ± 0.03	−39.4 ± 3.5	7.2 ± 0.1	0.8 ± 0.1
B9	5	10	240.0 ± 2.7	0.63 ± 0.01	−44.4 ± 4.2	14.2 ± 1.2	1.6 ± 0.1

*n = 3, All formulae contained 100 mg drug and 300 mg l-α-Phosphatidylcholine.

### 2.4. Characterization of the prepared sofosbuvir bilosomes

#### 2.4.1. Analysis of vesicular size, polydispersity index and zeta potential

Dynamic light scattering adopted in the Zetasizer (Nano ZS, Malvern Instruments, Malvern, UK) was utilized for the analysis of the VS, PDI and ZP of the bilosomal formulae. Samples taken from each formula were diluted with distilled water until being hazy (1:10 v/v dilution) before analysis.

#### 2.4.2. Determination of the encapsulation efficiency and the drug loading of the prepared sofosbuvir bilosomes

Sofosbuvir-loaded bilosomes were separated from the un-encapsulated drug by centrifugation at a speed of 15,000 rpm for 1 h and a temperature of 4 °C using a high-speed cooling centrifuge (Andreas Hettich GmbH and Co. KG, Tuttlingen, Germany). The supernatant of each sample was analyzed using UV-spectrophotometer (Shimadzu, Tokyo, Japan) at λ_max_ of 262 nm to determine the sofosbuvir concentration. The encapsulation efficiency was calculated using the following formula:

(1) $$\mathrm{EE}=\;\frac{\text{total drug amount}-\text{unentrapped drug in the supernatant}}{\text{total drug amount }}\;\times \;100$$

Moreover, the drug loading percentages (DL) of the prepared bilosomal formulae were calculated according to the following equation:

(2) $$\mathrm{DL}=\frac{\text{total drug amount encapsulated in the formula}}{\text{total formula weight}}\times \;100$$

#### 2.4.3. Optimization of the prepared bilosomal formulae

The desirability of the prepared formulae was calculated using Design-Expert^®^ software (Version 7, Stat-Ease Inc., Minneapolis, MN) and considered to optimize the studied responses according to the required constraints (Nour et al., 2016). The significant responses were taken into considerations while the non-significant factors were not. The bilosomes formula with the highest desirability value (close to 1) was taken for further investigation.

### 2.5. Freeze-drying of the optimized sofosbuvir bilosomes

Different cryoprotectants (mannitol and trehalose, each with a concentration of 2.5 or 5%) were added to samples of the optimized bilosomal formula before freeze-drying. The optimized bilosomal formula was frozen overnight at −20 °C and then, freeze-dried for 24 h using a lyophilizer (Novalyphe-NL 500; Savant Instruments Corp., Holbrook, NY, USA). The pressure was maintained at 7 × 10^−2^ mbar while the condenser temperature was kept at −45 °C for 24 h. The freeze-drying effect in the presence and absence of each cryoprotectant was investigated through measuring the VS, PDI, ZP and EE after reconstitution of the freeze-dried formulae and comparing the obtained data to the respective values measured before freeze-drying.

### 2.6. Molecular docking studies

Molecular Operating Environment (MOE program 2008.10; Chemical Computing Group, Montreal, QC, Canada) was used to perform the molecular docking studies. The crystal structures of ASBT taurocholate (PDB: 3ZUY) and ASGPR carbohydrate-binding site (PDB: 5JPV) were downloaded from the RSCB protein databank (Hu et al., 2011; Sanhueza et al., 2017). The downloaded crystal structures were prepared for docking by adding the missing protons, deleting any unnecessary co-crystallized ligands and metals using MOE software. For the ASBT transporter, pharmacophore placement was used. The pharmacophore was identified with the 3α- and 7α-hydroxyl groups as donors or acceptors, and the sulfonate was selected as the third group in the pharmacophore. The docking parameters that were used are ASE for rescoring 1 with 30 retained poses, Forcefield refinement, and London dG for rescoring 2 with 30 retained poses. For ASGPR transporter, pharmacophore placement was also used. The pharmacophore was identified with the 2-, 3-, and 4-hydroxyl groups of galactose molecules in the co-crystallized lactose as hydrogen bond donors or acceptors. The docking parameters that were used are London dG for rescoring 1 with 30 retained poses and Forcefield refinement with 30 retained poses. In both cases, the best poses in terms of docking scores and binding interactions to the active site are discussed in the text.

### 2.7. Synthesis, preparation and characterization of galactose-anchored bilosomal formula

Galactosylated taurocholate was synthesized through a one-pot acid-catalyzed condensation reaction of amide and aldehyde (STC and d-galactose) (Noyes & Forman, 1933; Milenkovic et al., 1999; Jacobi von Wangelin et al., 2003). In a Dean-Stark water trap, 1.2 g of STC was allowed to react with 1 g of d-galactose in 70 ml xylene in pH 4 adjusted using HCl for 7 h at 110 °C. At the end of the7 h, a dark brown precipitate was formed. The precipitate was washed three times with ethanol and distilled water, then, saved for further reactions and analysis. Fourier Transform Infrared (FTIR) spectra of STC, galactosylated taurocholate were recorded on KBr pellets with an FTIR spectrophotometer (Nicolet iS10, Thermo Fisher Scientific, Waltham, MA, USA). ^1^H NMR was performed on a 400 MHz spectrometer (Bruker LLC, Billerica, MA, USA) using DMSO-d6 as a solvent for both STC and galactosylated taurocholate. The synthesized galactosylated taurocholate was utilized to re-prepare the optimized bilosomal formula using the same technique and then, characterization parameters were repeated to study the effect of the applied chemical modification.

### 2.8. Imaging of the optimized formula

One drop of optimized bilosomal formula was placed on a grid of carbon-coated copper followed by adding a drop of 1% phosphotungstic acid solution. Samples were dried at room temperature and then, visualized using a transmission electron microscope (JEOL, Tokyo, Japan) at 100 kV. On the other hand, samples from the lyophilized optimized formula were gold-coated under vacuum and then, inspected using a scanning electron microscope (JXA-840; JEOL, Tokyo Japan).

### 2.9. *In vitro* drug release from the optimized bilosomal formula

Sofosbuvir release from the optimized bilosomal formulae before and after lyophilization and galactosylation was determined using the reverse dialysis technique in the USP II dissolution apparatus (Pharm Test, Hainburg, Germany) (Zhuang et al., 2010). The used dissolution medium was composed of 900 mL 0.1 N HCl (pH 1.2) for 2 h, then phosphate buffer saline (pH 6.8) for the rest of the 8 h. Dialysis bags (molecular weight cut off 12–14 kDa) were filled by 3 ml of the dissolution medium and an amount of each formula equivalent to 30 mg drug. The sofosbuvir release rate from its equivalent aqueous solution was taken as a reference. The rotation speed was adjusted to 50 rpm and the temperature was set at 37 ± 1 °C. Samples were taken at the following time intervals: 0.25, 0.5, 1, 2, 3, 4, 6 and 8 h. The drug concentration was spectrophotometrically analyzed at the predetermined **λ**_max_ (262 nm).

### 2.10. *In vivo* study

The protocol of the study was reviewed and approved by the institutional review board of the Faculty of Pharmacy, Cairo University (PI 2398). Eighty-one BALB/c albino mice were used for investigation of the *in vivo* behavior of the optimized formulae. Mice were housed under normal environmental conditions (room temperature 25 °C ± 0.5 and relative humidity of 65%) with free access to standard mice diet and water. At the study day, the animals were randomly divided into three groups, group I administered the lyophilized optimized bilosomal formula (LOBF), group II took the galactose-anchored bilosomal formula (Galactosylated LOBF) and group III administered the drug solution, each in an amount equivalent to 60 mg/kgBW (Reagan-Shaw et al., 2008). All the groups received the formulae and the drug solution orally. Three mice from each group were sacrificed after 0.25, 0.50, 1, 2, 3, 4, 6, 8 and 24 h following the administration. The liver of each mouse was separated and homogenized with 3 mL normal saline. The homogenate was directly transferred into plastic tubes and stored at −70 °C until the analysis time. Tadalafil (100 µL; 100 ng/mL) was added to each sample as an internal standard. A liquid/liquid extraction technique was adopted using methyl *tert*-butyl methyl ether which was added to the samples in a ratio of 7:1 v/v, respectively. The organic solvent is vortexed for 5 min with the samples, centrifuged at 5000 rpm and then, evaporated under vacuum at a temperature of 50 °C. A sensitive, selective and accurate LC-MS/MS method (API-4000, AB Sciex, Foster, CA, USA) was developed and validated before the study for the determination of sofosbuvir. The mobile phase composed of acetonitrile: ammonium formate buffer (5 mmoL, pH 3.5), in a ratio of 1:1 v/v, running at a rate of 0.7 mL/min. A Zorbax Eclipse-Plus column from Agilent, Santa Clara, CA, USA (4.6 × 50 mm; 5 µm) was utilized to separate sofosbuvir and tadalafil. Finally, the pharmacokinetic parameters were determined using non-compartmental pharmacokinetic models using Kinetica^®^ software version 5 (Thermo Fisher Scientific Inc., Waltham, MA, USA). Both drug targeting index (DTI) at time 6 h and relative targeting efficiency (RTE) were calculated according to the following equations:

(3) $$\mathrm{DTI}=\;\frac{\text{liver conc}.\text{after oral administeration of nanovescilces at time }(t)}{\text{liver conc}.\text{after oral administeration of drug solution at time }(t)}$$

(4) $$\mathrm{RTE}=\;\frac{\text{liver }{\mathrm{AUC}}_{\left(0-24\right)}\text{after oral administeration of nanovescilces}}{\mathrm{liver}\;{\mathrm{AUC}}_{\left(0-24\right)}\text{after oral administeration of drug solution}}$$

## 3. Results and discussion

### 3.1. Characterization of the prepared sofosbuvir bilosomes

#### 3.1.1. Analysis of vesicular size, polydispersity index and zeta potential

VS of the prepared sofosbuvir nanovesicles was fairly low within a range of 149–240 nm (Table 1). As the size is below 300 nm, this could be useful in enhancing the gastrointestinal transport through enterocytes and M cells to the systemic circulation as concluded before by He *et al* (2012). The VS values were statistically analyzed according to a linear model. The model was significantly expressing the data (*p*-value = 0.0007) with a non-significant lack of fit (*p*-value = 0.5625) and an acceptable signal to noise ratio (11.65). Moreover, the model predicted R^2^ (0.5869) was in harmony with the adjusted R^2^ (0.7169) with a difference of less than 0.2 (Raissi & Farsani, 2009). The following equation describes the effects of the studied factors on the VS values:

(5) $$Y_{1}=201.15+31.15X_{1}-7.42X_{2}$$

**Figure 1.**
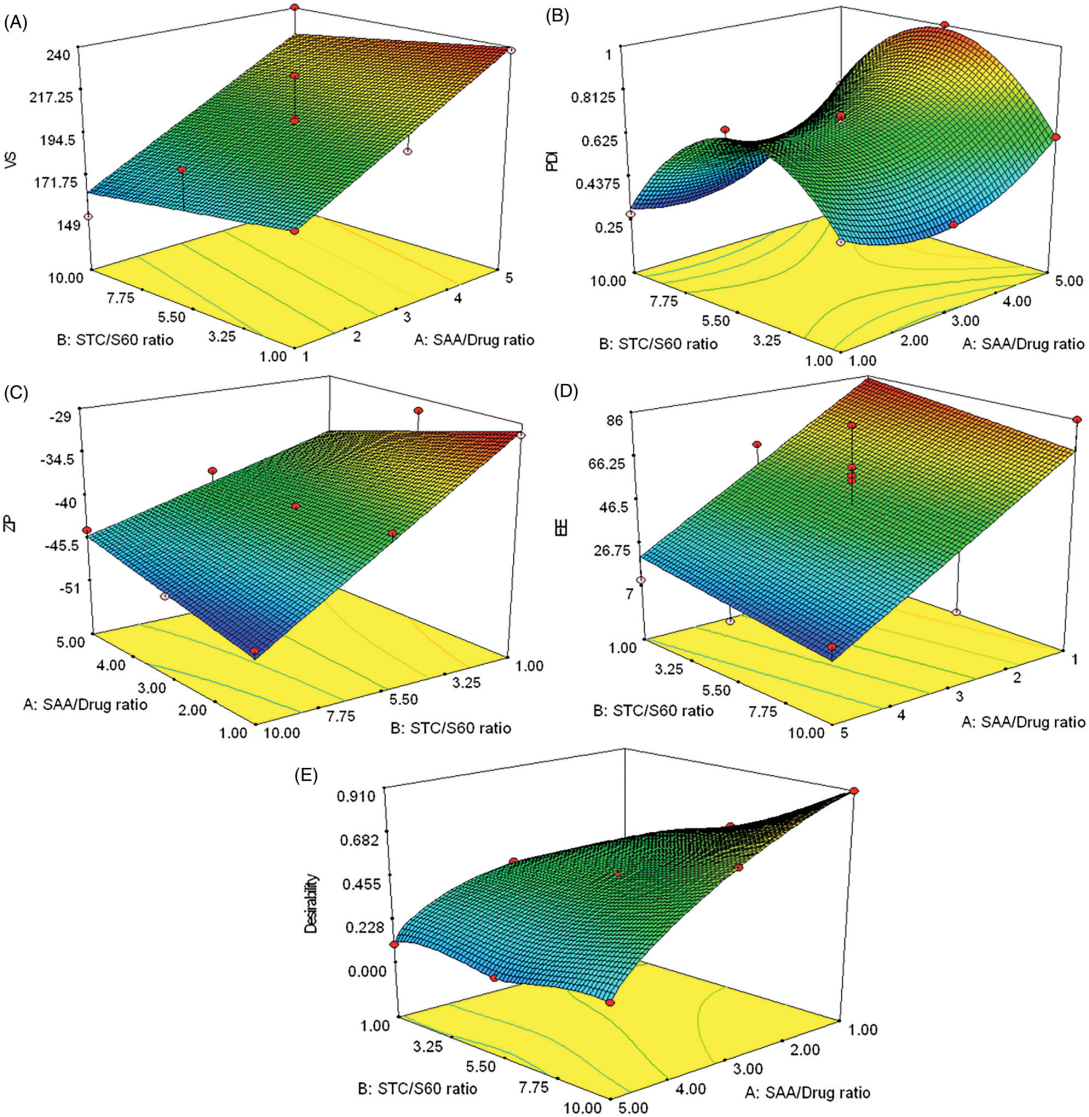
Response surface plot for the effects of SAA to drug ratio (X_1_) and STC to S60 ratio (X_2_) on the particle size (A), polydispersity index (B) zeta potential (C), entrapment efficiency (D) and desirability (E) of sofosbuvir bilosomal formulae.

The VS was significantly affected by the SAA to drug ratio (*p*-value = 0.0002), where increasing the SAA led to a significant increase in the VS, as shown in Table 1 and Figure 1(A). This could be attributed to the relatively high SAA concentrations utilized in the preparation of sofosbuvir bilosomes which might form bigger aggregates upon increasing its ratio to the drug. Similar studies reported the increase in particle size once the SAA concentration exceeded 50% weight of the prepared system (Kommuru et al., 2001; Wang et al., 2009; Yoo et al., 2010; Saberi et al., 2013). The reason behind the nanovesicular aggregation could be also referred to as the affinity of the STC to form dimers at high concentration and the hydrophobicity of S60 which led to growth in the size of the already formed nanovesicles rather than forming new ones (Bottari et al., 1999; Zhou et al., 2007).

PDI of the bilosomal formulae was scattered over a wide range starting from 0.271 and ending with 1, as demonstrated in Table 1 and Figure 1(B). The effects of SAA to drug ratio and STC to S60 ratio were analyzed according to a quadratic model which could significantly navigate the design space (*p*-value < .0001) with a non-significant lack of fit (*p*-value = .2676), relatively high signal to noise ratio (27.931) and coincided R^2^ values (Predicted *R*^2^ = 0.8997 and adjusted R^2^ = 0.9665). The multivariant equation was as follows:

(6) $$Y_{2}=0.69+0.13X_{1}-0.031X_{2}+0.049X_{1}X_{2}+0.17X_{1}^{2}-0.35X_{2}^{2}$$

PDI was directly proportional to the SAA to drug ratio with a *p*-value of less than 0.0001. This might be correlated to the gradual aggregation that took place with the addition of more SAA. The more SAA was added, the more variability in size between the aggregated and the non-aggregated nanovesicles. Additionally, each of the utilized SAA had an affinity to form aggregates of different shapes and sizes; i.e. STC and S60 formed dimers and hexagonal clusters, respectively (Bottari et al., 1999; Zhou et al., 2007). Additionally, excess SAA could form micelles with very small VS, compared to bilosomes, leading to a significant size variability and consequently, an increase in the PDI values.

High ZP was observed on the surface of the prepared bilosomal formulae, ranged between −30.6 mV and −49.5 mV, as shown in Table 1 and Figure 1(C). This range could guarantee high physical stability for the bilosomes over the long term due to the significant repulsion between the similarly charged surfaces (Chun et al., 2017; Sala et al., 2017). A two-factor interaction model was adopted to analyze the measured ZP values. The model’s lack of fit was not significant (*p*-value = 0.1014) and the model prediction capabilities were high with a predicted R^2^ (0.8780) close to the adjusted R^2^ (0.9283). The following equation shows the significance of each studied parameter on changing the ZP:

(7) $$Y_{3}=-40.33-0.64X_{1}-7.01X_{2}+2.46X_{1}X_{2}$$

Increasing the STC ratio led to a statistically significant increase in the ZP in the negative direction with a *p*-value of less than 0.0001. This could be referred to as the anionic nature of STC which imparted a negative potential on the bilosomal surfaces (Qiao et al., 2018; Ahad et al., 2018).

#### 3.1.2. Determination of the encapsulation efficiency and the drug loading of the prepared sofosbuvir bilosomes

Sofosbuvir prepared bilosomes had a variable EE in the prepared bilosomal formulae ranged from 7.2% to 85.8%, as displayed in Table 1 and Figure 1(D). The EE findings were statistically analyzed using a factorial ANOVA with a linear model equation:

(8) $$Y_{4}=46.59-32.17X_{1}-6.15X_{2}$$

The linear model was validated based on its adequate precision (7.45) and the values of the adjusted and the predicted R^2^ (0.5011 and 0.3908, respectively). Moreover, the lack of fit for this linear model was statistically non-significant with a *p*-value of .7865. Increasing the SAA to drug ratio had a significantly negative effect on the EE (*p*-value = 0.0042). This could be related to the drug micellar solubilization that took place at higher SAA concentrations. Sofosbuvir might escape from the bilosomal nanovesicles to the mixed micelles formed of STC and S60 only without PC. Similar observations were reported by El-Samaligy et al. (2006) who studied the effect of increasing Tween 20 and Tween 80 on Silymarin encapsulation in the prepared liposomal vesicles.

On the other hand, the drug loading in the bilosomal formulae ranged from 0.8% to 17.2%, as shown in Table 1.

#### 3.1.3. Optimization of the prepared sofosbuvir bilosomes

The optimized composition of the prepared bilosomes was determined based on the desirability equation taking into consideration achieving the least VS, PDI, the highest ZP and EE. The formula B3, composed of SAA to drug ratio of 1:1 w/w and sodium taurocholate to span ratio of 10:1 w/w, had the highest desirability value (0.908) with a VS, PDI, ZP and EE of 149 nm, 0.271, −49.5 mV and 85.85%, respectively, as shown in Table 1 and Figure 1(E). The optimized formula; nominated as the optimized bilosomal formula (OBF), was prepared again and subjected to further physical and chemical modifications, in addition to *in vitro* and *in vivo* investigations.

### 3.2. Characterization of the lyophilized sofosbuvir bilosomes

The lyophilized optimized formula maintained its original VS, ZP and EE measured before freeze-drying in the absence of any cryoprotectant, with no statistically significant difference (*p*-values = 0.1032, 0.0718 and 0.1843, respectively). Consequently, there was no need for cryoprotectant in terms of the obtained VS values. This might be attributed to the high zeta potential (≈−50 mV, as shown in Table 2) which could stabilize the bilosomes and prevent its aggregation during freeze-drying (Han et al., 2013). On the other hand, PDI significantly increased to 0.43 and this could be referred to as the incomplete reconstitution of the freeze-dried components leading to the presence of few aggregates with relatively higher VS (Moretton et al., 2012; Ball et al., 2017). Unfortunately, PDI values were not improved in the presence of any of the utilized cryoprotectants. Similar results were obtained by Doktorovova et al. (2014) while studying the effect of trehalose as a cryoprotectant for the freeze-drying of solid lipid nanoparticle formulation. Based on these findings, the lyophilized optimized liposomal formula (LOBF) without a cryoprotectant was adopted to proceed with further development and characterization.

**Table 2.** Particle size, polydispersity index, zeta potential and entrapment efficiency of the lyophilized formulae, before and after galactosylation.

Formula code	Type of cryoprotectant	Percentage of cryoprotectant (%)	VS (nm)	PDI	ZP(mV)	EE (%)
L1	–	–	141.3 ± 7.9	0.43 ± 0.03	−51.1 ± 1.6	83.1
L2	Mannitol	2.5	186.8 ± 5.7	0.53 ± 0.04	−54.3 ± 1.2	84.6
L3		5	177.2 ± 1.4	0.49 ± 0.03	−54.2 ± 2.5	82.0
L4	Trehalose	2.5	162.5 ± 4.8	0.51 ± 0.01	−55.5 ± 4.2	86.2
L5		5	174.9 ± 3.3	0.49 ± 0.02	−55.4 ± 1.9	83.9
Galactosylated LOBF	–	–	148.9 ± 3.0	0.48 ± 0.05	−53.7 ± 3.9	85.3

*n = 3.

### 3.3. Design of the galactosylated taurocholate conjugate and molecular docking studies

#### 3.3.1. Design of the galactosylated taurocholate conjugate

**Figure 2.**
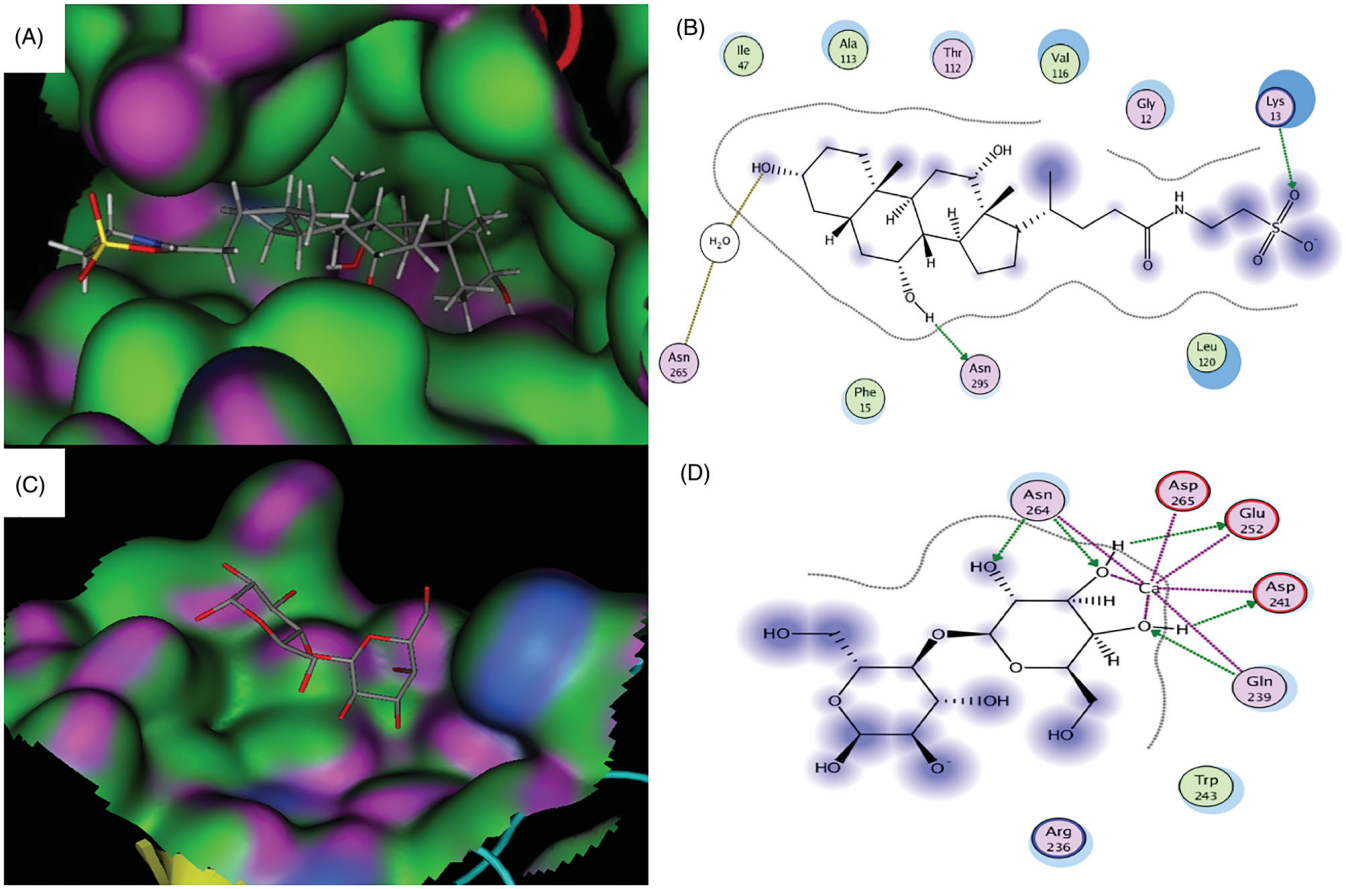
(A) 3D binding and (B) 2D binding interactions of taurocholate to the ASBT taurocholate-binding site. (C) 3D binding and (C) 2D binding interactions of the co-crystallized lactose with the ASGPR carbohydrate-binding site. For A and C: Green = hydrophobic surface, purple = hydrogen bonding site, and blue = mild polar. Atoms color coding: gray = carbon, red = oxygen, blue = nitrogen, yellow = sulfur. For B and D: Hydrogen bonds and bonds to metals are shown as blue and green dashed arrows. Hydrogen bonds through water bridges are represented as brown dotted lines. Amino acids spheres: pink circled in red = acidic, pink circled in blue = basic, pink circled in black = polar, green circled in black = greasy.

The ASBT taurocholate binding site is a deep pocket that could accommodate taurocholate and other bigger compounds (Hu et al., 2011). The pocket is characterized by having several hydrophobic amino acids lining the binding site with a wide active site entrance (Figure 2(A)). According to the crystal structure of the ASBT taurocholate binding site, there is one key hydrogen bonding interaction between the 3α-hydroxyl group and Asn265 toward the bottom of the binding site (Figure 2(B)). Besides, there is another important interaction between the 7α-hydroxyl group with Asn295 through a water bridge (Hu et al., 2011). These interactions seem to be crucial for the binding affinity and the uptake of taurocholate by the ASBT transporter. A previous study reported that mutations of Asn295 or Asn265 to alanine significantly reduced the transporter activity and taurocholate uptake by around 70–80% which showed the importance of the two amino acids residues and the two hydrogen bonding interactions for efficient taurocholate uptake by ASBT transporter (Hu et al., 2011). On the other hand, the carbohydrate-binding site of the ASGPR co-crystallized with lactose showed that it is shallow and more open than ASBT, and it could accommodate bigger molecules with no steric clashes (Figure 2(C)) (Sanhueza et al., 2017). Also, several key interactions were found between hydroxyl groups of the galactose molecule and the calcium ion in the binding site and other amino acids (Gln239, Asp241, Glu252, and Asn264) (Figure 2(D)) (Sanhueza et al., 2017).

The nature of both ASBT taurocholate-binding site and ASGPR carbohydrate-binding site allowed us to design a conjugate that would fit and bind to both active sites with no steric clashes or loss of the crucial interactions with each site. The wide active site entrance along with the hydrophobic and deep nature of the ASBT taurocholate pocket, shown in Figure 2(A), would allow attaching the galactose molecule to the side chain of taurocholate and not the steroid nucleus or the 3α- or 7α-hydroxyl groups of taurocholate. Conjugating taurocholate and galactose in this way could avoid any steric clashes of the galactosylated taurocholate conjugate with the binding site of taurocholate, and it would position the galactose fragment of the conjugate toward the solvent-exposed region of the ASBT taurocholate binding site (Figure 2(A)). Besides, keeping the 3α- and 7α-hydroxyl groups of galactosylated taurocholate conjugate free would avoid losing any of the key binding interactions between the 3α- and 7α-hydroxyl groups with Asn265 and Asn295 that are essential for the efficient uptake of taurocholate (Figure 2(B)) (Hu et al., 2011).

Molecular docking studies were done to simulate the binding of galactosylated taurocholate conjugate to both ASBT taurocholate (PDB: 3ZUY) and ASGPR carbohydrate (PDB: 5JPV) binding sites. The molecular docking studies aimed to ensure that there are no unfavorable interactions between galactosylated taurocholate conjugate and the binding sites and that connecting galactose and taurocholate in such a way would not compromise the key interactions for binding and uptake mentioned above.

#### 3.3.2. Molecular docking with ASBT taurocholate binding site

**Figure 3.**
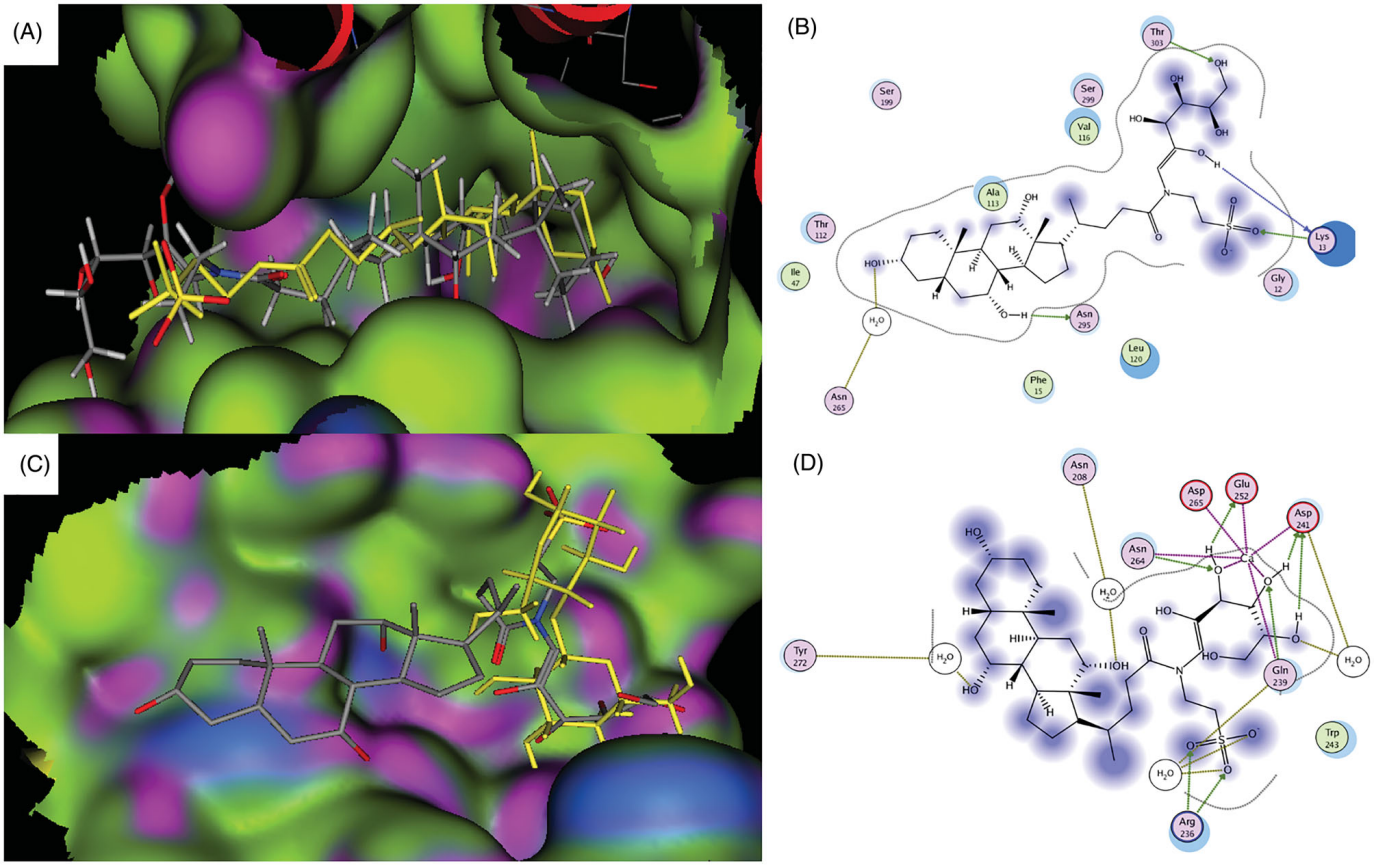
Docking pose of the galactosylated taurocholateconjugate bound to ASBT taurocholate (A, B) and ASGPR carbohydrate (C, D) binding sites. For color coding, see Figure 2.

To validate the docking procedure, the docking of the unsubstituted taurocholate was done. Taurocholate showed the expected binding with the known two interactions between the 3α-hydroxyl and Asn265 through a water bridge, and between and the 7α-hydroxyl and Asn295 (Figure 2(B)) (Hu et al., 2011). The calculated docking score of taurocholate was −15.5249 kcal/mol. Galactosylated taurocholate conjugate was then docked into the binding site using the same docking parameters (Figure 3(A,B)). The conjugate displayed good superimposition with the co-crystallized taurocholate ligand in the binding site of ASBT transporter, where the steroid backbone was embedded in the active site and the side chain with the conjugated galactose was placed toward the solvent-exposed region of the pocket (Figure 3(A)). The two known critical hydrogen-bonding interactions were also observed, as shown in Figure 3(B). Interestingly, two hydroxyl groups of the conjugated galactose formed two additional hydrogen-bonding interactions with Lys13 and Thr303. The docking score for the conjugate was −17.7755 kcal/mol. The additional hydrogen bonding interactions and lower docking score suggested that the conjugate molecule had a stronger affinity to the binding site of the transporter compared to taurocholate, and it could show better uptake by ASBT than taurocholate.

#### 3.3.3. Molecular docking with ASGPR carbohydrate-binding site

Galactose was docked into the ASGPR carbohydrate-binding site to validate the docking parameters. As expected, the molecule was perfectly positioned in the shallow binding site showing the reported interactions with the calcium ion and several hydrogen bonds with Gln239, Asp241, Glu252, and Asn264 with −17.8014 kcal/mol docking score (Sanhueza et al., 2017). The docked galactosylated taurocholate conjugate exhibited superimposition of the galactose part of the conjugate with the galactose part of the co-crystallized lactose molecule, while the taurocholate fragment of the conjugate molecule was placed toward the solvent-exposed region (Figure 3(C)). Several binding interactions were also observed with the calcium ion and Gln239, Asp241, Glu252, and Asn264 (Figure 3(D)). Also, the taurocholate fragment showed several additional binding interactions either by direct interactions or through water bridges (Figure 3(D)). The additional observed interactions were between the 7α-hydroxyl and Tyr272, the 12α-hydroxyl and Asn208, and the sulfonate group with Arg236 and Gln239 (Figure 3(D)). The docking score for the observed docking pose was −22.6783, which suggested along with the additional binding interactions that the galactosylated taurocholate conjugate had higher binding affinity that galactose and could have better uptake efficiency than galactose.

The overall outcome of the molecular docking studies supported the design approach. The molecular docking studies also showed that the binding of the galactosylated taurocholate conjugate to both transporters was not affected and that all the essential binding interactions were observed with both binding sites of ASBT and ASGPR transporters.

### 3.4. Synthesis, preparation and characterization of the galactose-anchored bilosomal formula

#### 3.4.1. Reaction mechanism

**Figure 4.**
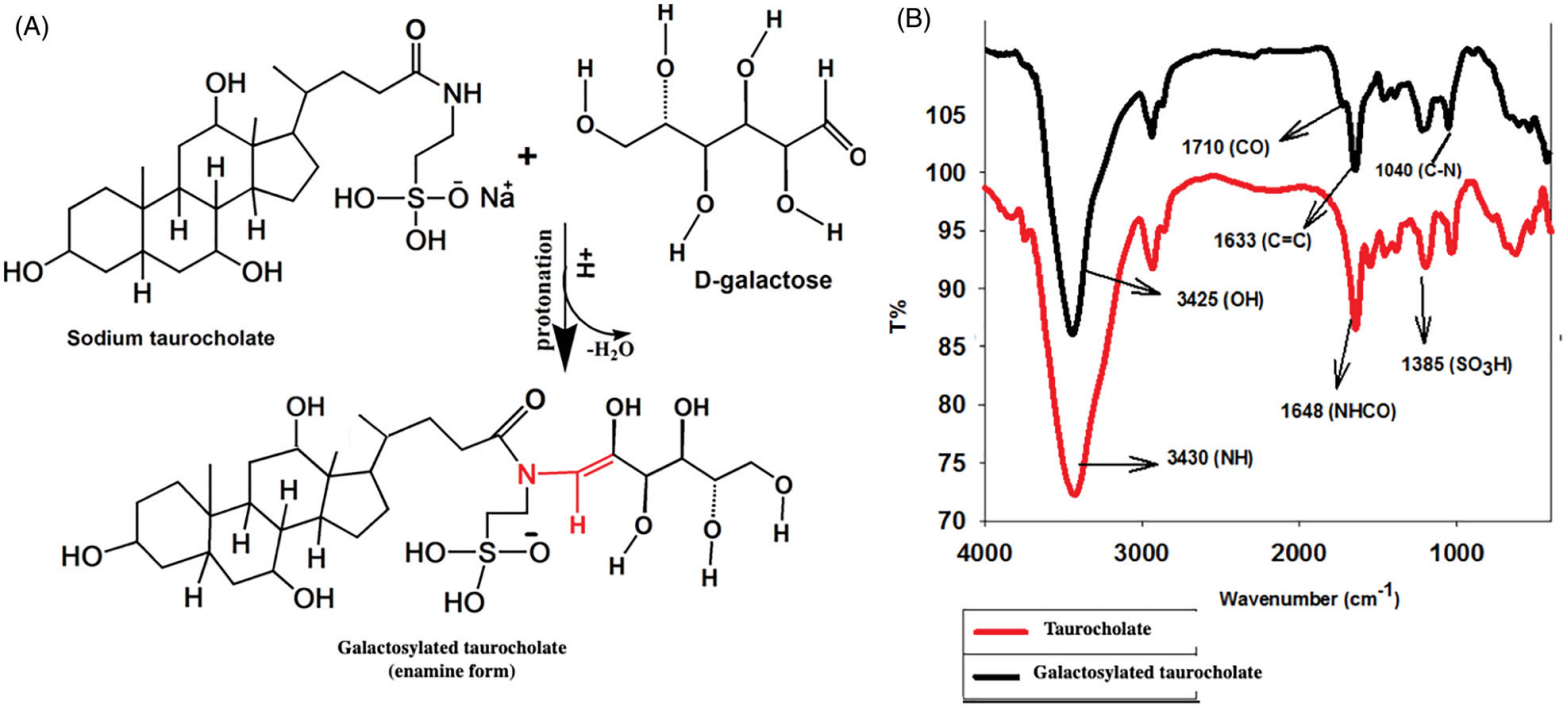
Mechanism of galactosylation of sodium taurocholate (A) and the FTIR spectra of sodium taurocholate and galactosylated taurocholate (B).

STC as a secondary amide and is considered as non-basic non-acidic molecule under physiological conditions. In the presence of very strong acid (HCl) and high temperature (110°C), amides are partially protonated. This reactivity is taken as an advantage in condensation and hydrolysis reactions (DeRuiter, 2005; Mesher et al., 2017). On the other hand, d-galactose in acidic solution transforms into an enol tautomeric structure which promotes removing a water molecule during the reaction between STC and d-galactose to form the enamine form of galactosylated taurocholate, as shown in Figure 4(A) (Cook, 2017).

#### 3.4.2. FTIR spectra studies

FTIR spectra confirmed the formation of galactosylated taurocholate, as shown in Figure 4(B). The characteristic peaks of enamine appear at 1633 cm^−1^ refer to C=C of galactosylated taurocholate. Also when comparing galactosylated taurocholate FTIR spectra with that of STC, a new peak was formed at 1710 cm^−1^ which refers to the formation of true ketone (C=O) during the reaction between d-galactose and taurocholate, as shown in Figure 4(A)(Adams, 2000; Patze et al., 2011). The peak of the taurocholate N–H group changed from broadband at 3430 cm^−1^ to a sharp one 3425 cm^−1^. This might prove the formation of galactosylated taurocholate through keto-enol reaction which removed a hydrogen atom of the N–H group to form tertiary amine group (N) and also prove the entrance of OH groups of d-galactose in the taurocholate structure. Moreover, a new peak appeared at 1041 cm^−1^which might refer to the C–N bond of enamine in the galactosylated taurocholate structure. A peak at 1383 cm^−1^ in both taurocholate and galactosylated taurocholate could be attributed to the sulfonate group peak (O=S=O). From all the above, galactosylated taurocholate synthesized by acidic condensation reaction to form a stable enamine compound.

#### 3.4.3. Nuclear magnetic resonance (^1^H NMR) studies

**Figure 5.**
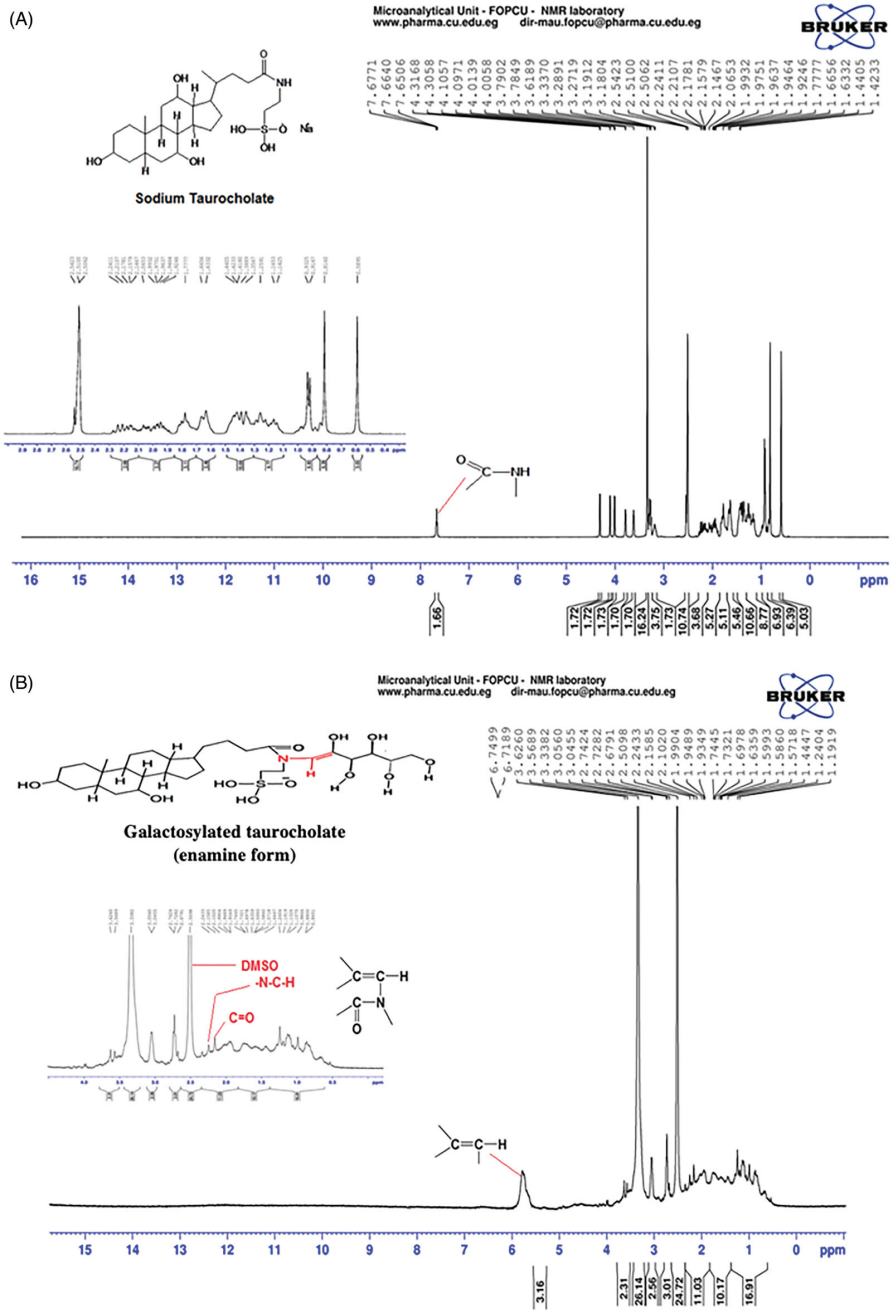
^1^HNMR spectrum of Sodium taurocholate (A)and galactosylated taurocholate (B) in DMSO.

STC and galactosylated taurocholate were characterized by ^1^H NMR. As shown in Figure 5(A), STC had a strong characteristic peak at 6.8 ppm referring to the H atom of the STC amide group (H–N–C=O). This peak disappeared in ^1^H NMR spectra of galactosylated taurocholate (Figure 5(B)) and another one appeared at 5.8 ppm referring to H attached to the conjugated vinylic group (–C=C–H) which is the main characteristic peak for enamine compounds. Moreover, peaks at 2.1, 2.6 and 2.7 ppm could refer to the OH groups of d-Galactose attached to taurocholate. The peak at 2.3 could be attributed to the C=O group of the ketone form. The absence of the 9.6 ppm peak of aldehyde hydrogen atom might prove the keto-enol reaction, suggested in Figure 4(A). Also, the peak at 3.3 ppm might refer to (–N–C–H) group. Consequently, H^1^NMR spectra proved the formation of galactosylated taurocholate as enamine; and these findings were in agreement with FTIR spectra, shown in Figure 4(B).

#### 3.4.4. Characterization of galactose-anchored bilosomal formula

The galactosylated bilosomal formula had VS, PDI, ZP and EE values near to the optimized bilosomal formula, either before (OBF) or after (LOBF) lyophilization with *p*-values > 0.05, as shown in Table 2.

### 3.5. Imaging of the optimized formula

**Figure 6.**
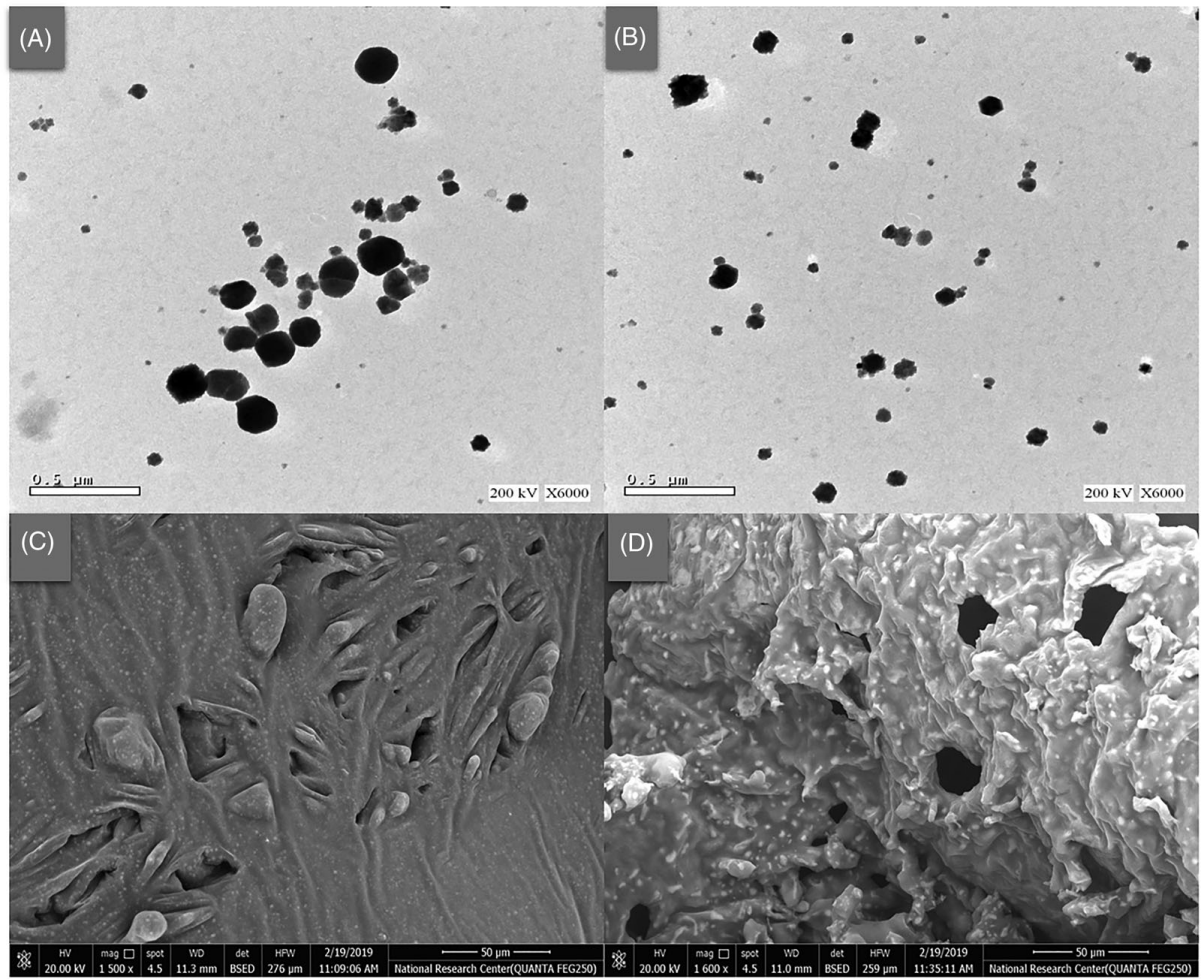
Transmission (X 6000) and scanning (X 1500) electron micrographs of the optimized bilosomal formula (OBF), before (A, C) and after galactosylation (B, D), respectively.

The prepared bilosomes appeared under the TEM as spheres with an incompletely regular outline and relatively rough surfaces, as shown in Figure 6(A,B). Sizes of the imaged bilosomes, before and after galactosylation, were matching with the values measured by the Zetasizer for the same formula (OBF). On the other hand, relatively homogenous and slightly porous matrices were formed after lyophilization of the optimized formula, either before or after galactosylation, as demonstrated in the SEM micrographs (Figure 6(C,D)).

### 3.6. *In vitro* drug release from the optimized bilosomal formula

**Figure 7.**
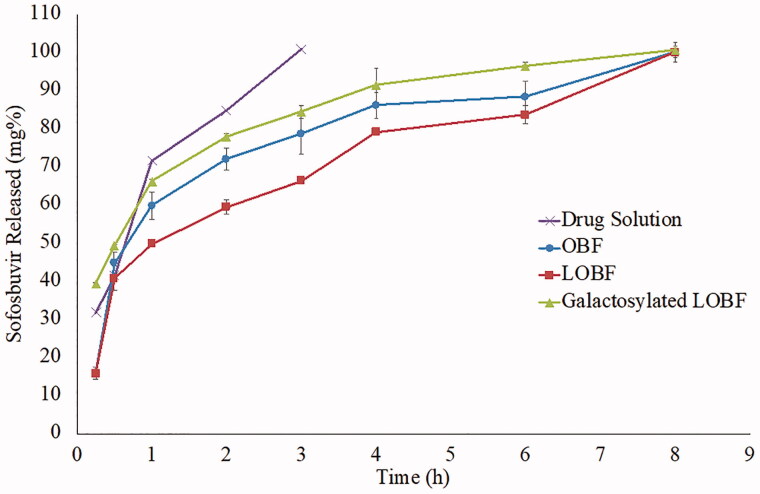
Drug release profiles from the optimized bilosomal formula as a dispersion (OBF) and lyophilized powder (LOBF), before and after galactosylation, in comparison with the drug solution.

Reverse dialysis technique was adopted to ensure that the dissolution environment simulates the physiological sink conditions and prevent physical aggregation of the tested bilosomes with the limited space inside the dialysis bag (Amatya et al., 2013). Sofosbuvir was fully released within 3 and 8 h from the drug solution and the optimized formulae, respectively, as demonstrated in Figure 7. The release was following the Higuchi diffusion model with R^2^ values of 0.9645, 0.8874, 0.9433 and 0.9975 for the drug solution, OBF, LOBF and the galactosylated LOBF, respectively. T_50%_ of the drug release was 0.78 h in case of the drug solution while it was significantly higher in case of the optimized bilosomal formula, OBF (1.50 h, *p*-value < 0.0001) which could reflect the capability of the formula to keep most of the drug entrapped till reaching the target site. Additionally, LOBF had a relatively high T_50%_ (2.5 h) which could be referred to the expected lag time required to dissolve the lyophilized powder and reconstitute the bilosomes, when compared to the already dissolved drug molecules in case of the drug solution or the already formed bilosomes in case of the OBF formula (Webb et al., 2001). On the other hand, the galactosylated LOBF released the whole drug within 8 h with a T_50%_ of 1.04 h. The release rate was significantly higher than the non-galactosylated LOBF (*f2 =* 17) and this could be attributed to the galactose effect which increased the hydrophilicity of the bilosomal lipidic components (Kawakami et al., 1998; Lin & Chen, 2007).

### 3.7. *In vivo* study

**Figure 8.**
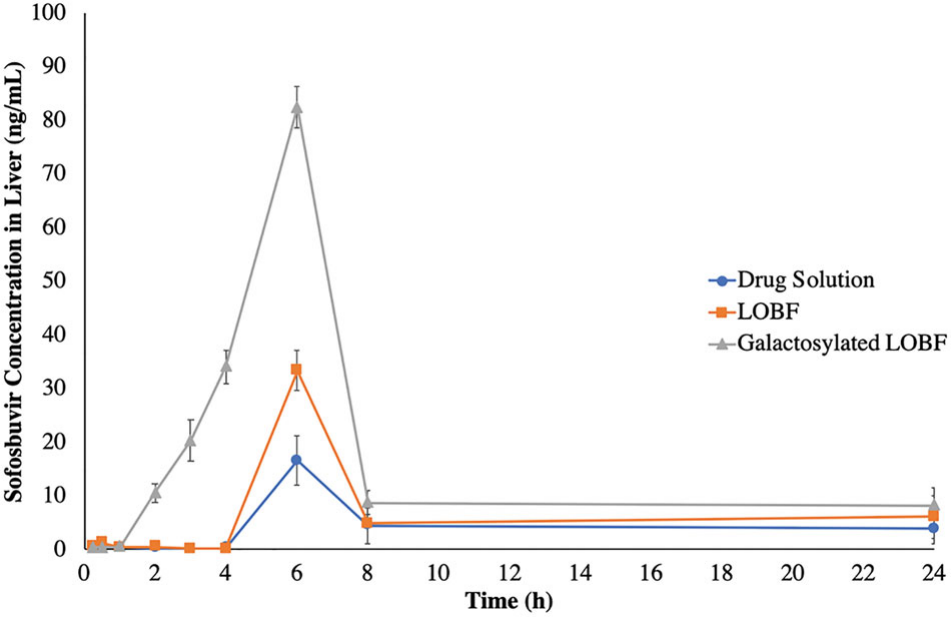
Mean sofosbuvir concentrations (ng/mL) in the liver after oral administration of the optimized bilosomal formula as a lyophilized powder (LOBF) before and after galactosylation, in comparison with the drug solution.

The utilized analytical method was validated in terms of linearity (R^2^ = 0.9987) within the range of 0.1–800 ng/mL, accuracy (100% ± 15), lower limit of quantification (LLOQ = 0.1 ng/mL) with quality control samples of (QCL = 0.3 ng/mL, QCM = 400 ng/mL and QCH = 640 ng/mL). Sofosbuvir was detected in the liver after a lag period of 4 h, as shown in Figure 8, after oral administration of either the drug solution or the LOBF. On the other hand, the galactosylated LOBF exhibited a faster drug delivery with shorter lag time (1 h). The drug reached its maximum level in the liver after 6 h in all formulae/drug solution. On the other hand, the maximum levels were significantly varied where the drug solution had the lowest value (16.60 ng/ml), the LOBF had a significantly higher value (33.39 ng/mL) and the galactosylated LOBF had the highest value (82.39 ng/mL), as shown in Table 3. The same pattern was applied to RTE as the LOBF and the galactosylated LOBF had values of 1.51 and 3.66, respectively, when compared to the drug solution. The enhancement of drug availability in the liver could be referred to as the preferential hepatic uptake of STC present in both bilosomal formulae (Dawson, 2017; Slijepcevic & van de Graaf, 2017). Also, the increase in the hepatic drug availability could result from the enhancement of drug absorption due to the bilosomal preparation (VS ≈ 140 nm) that might facilitate the drug internalization and the presence of dual SAAs (STC and S60) that might fluidize the membrane lipidic components and consequently, facilitate drug permeation (Arzani et al., 2015; Moghimipour et al., 2015; Mohsen et al., 2017). The maximum sofosbuvir targeting was achieved in the case of the galactosylated LOBF with a DTI value of 5.03, compared to the drug solution. This could be attributed to the additional binding between galactose and asialoglycoproteins receptors predominately expressed on the liver parenchymal surfaces (Huang et al., 2017; Liu et al., 2017; Singh et al., 2017). Also, it can be observed in Figure 8 that galactose-anchored bilosomes reduced the lag time of the drug appearance in the liver to 1 h instead of being 4 h in the other formulae.

**Table 3.** Sofosbuvir pharmacokinetic parameters in the liver after oral administration of the plain and galactosylated optimized bilosomal formulae, in comparison with the drug solution.

Pharmacokinetics parameters	Treatment (mean ± SD)^a^		
	The drug solution	LOBF	Galactosylated LOBF
C_max_ (ng/ml)	16.59 ± 1.9	33.38 ± 2.8	83.38 ± 7.4
T_max_ (h)^b^	6	6	6
AUC_0–24_ (ng.h/ml)	106.99 ± 6.0	161.91 ± 10.6	392.36 ± 12.5
AUC_0–∞_ (ng.h/ml)	176.30 ± 9.47	266.40 ± 12.69	511.33 ± 10.52
% Hepatic availability	–	151.10	290.04
DTI (6 h)		2.01	5.03
RTE		1.51	3.66

^a^*n* = 27.

^b^Median values of T_max_ is displayed instead of the mean.

## 4. Conclusions

A central composite design was successfully applied to the optimized sofosbuvir bilosomes with minimized VS, PDI and maximized ZP and EE. The optimized formula was prepared using galactosylated taurocholate in an attempt to improve its liver targetability and then lyophilized. The developed bilosomes utilized two vectors for achieving liver targeting; galactose and bile salts. *In vivo* results showed the ability of the prepared formulae to increase drug availability in the target organ. The galactose-anchored and taurocholate-stabilized bilosomes were capable to significantly increase sofosbuvir hepatic availability, compared to the corresponding drug solution. The research findings could improve the healthcare outcome to a wide range of patients around the world as sofosbuvir is co-administered with several antiviral drugs to enhance the recovery percentages. Additionally, the same concept could be investigated in future research as a vector-mediated nanocarrier for anticancer drugs acting on the liver.
